# Supplementary material for: Cloning and characterization of the homoeologous genes for the Rec8-like meiotic cohesin in polyploid wheat
Source: BMC Plant Biol. 2018 Oct 11;18:224. doi: 10.1186/s12870-018-1442-y (PMC6180652; doi:10.1186/s12870-018-1442-y)
Supplement: Supplementary file 1 — Figure S1. Comparative analysis of the cohesin-like proteins. Figure S2. Identification of the BAC clones containing the homoeoalleles TtRec8-A1 and TtRec8-B1 in LDN. Figure S3. CDS alignment of the homoeoalleles TtRec8-A1, TtRec8-B1, and TaRec8-D1. Figure S4. Predicted protein sequences of the wheat Rec8 homoeoalleles. Figure S5. Verification of polypeptide pGEX-R26 by protein identification assay. Table S1. DNA primers and their sequences used in this study. Table S2. Comparative analysis of the predicted TtRec8-A1 protein with the cohesion proteins from other eukaryotic species. Table S3. Genbank accession numbers of the Rec8 orthologues involved in the phylogenetic analysis. (DOCX 2639 kb) [file 12870_2018_1442_MOESM1_ESM.docx]

**Figure S1.** **Comparative analysis of the cohesin-like proteins.**

Amino acid sequences of TtRec8 is aligned with *Brachypodium* *distachyon* sister chromatid cohesion 1 protein 1-like protein BdScc1L (GenBank Accession XP_003567819.1), maize absence of first division1 ZmAfd1 (GenBank Accession NP_001105829.1), rice Rad21/Rec8-like protein OsRad21 (GenBank Accession NP_001056426.1), *Arabidopsis thaliana* sister chromatid cohesion 1 protein 1 AtSyn1 (GenBank Accession NP_196168.1), *Drosophila melanogaster* Rad21 (GenBank Accession AAD33593.1), and *Xenopus laevis* Rad21 (GenBank Accession AAH97558.1). The black, grey, and white backgrounds indicate the similarity levels of amino acids. The green and red lines above the sequence demarcate the conserved domains pfam04825 (N-terminus) and pfam04824 (C-terminus), respectively. The blue box highlights the serine-rich regions conserved among plant cohesin proteins, and the orange boxes highlight the potential PEST motifs.

**
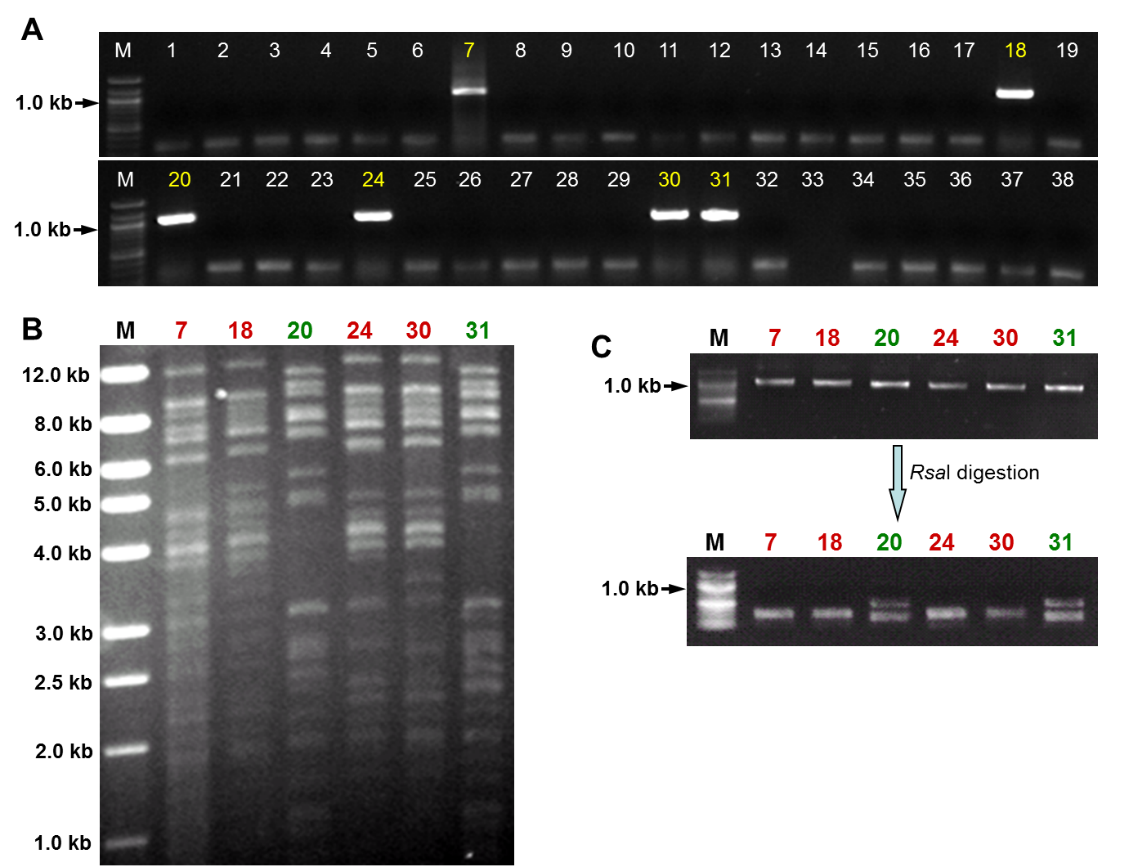
**

**Figure S2.** **Identification of the BAC clones containing the homoeoalleles *TtRec8-A1* and *TtRec8-B1* in LDN.**

**A)** Six positive BAC clones (No. 7, 18, 20, 24, 30, and 31) identified to contain the genomic DNA sequences of *TtRec8* by the *TtRec8*-specific primer pair GM008F/GM008R; **B)** *Hin*dIII-digested DNA fingerprinting of the six positive BAC clones; **C)** *Rsa*I-digested CAPS of the amplicons amplified by GM008F/GM008R in the six positive BAC clones.

**
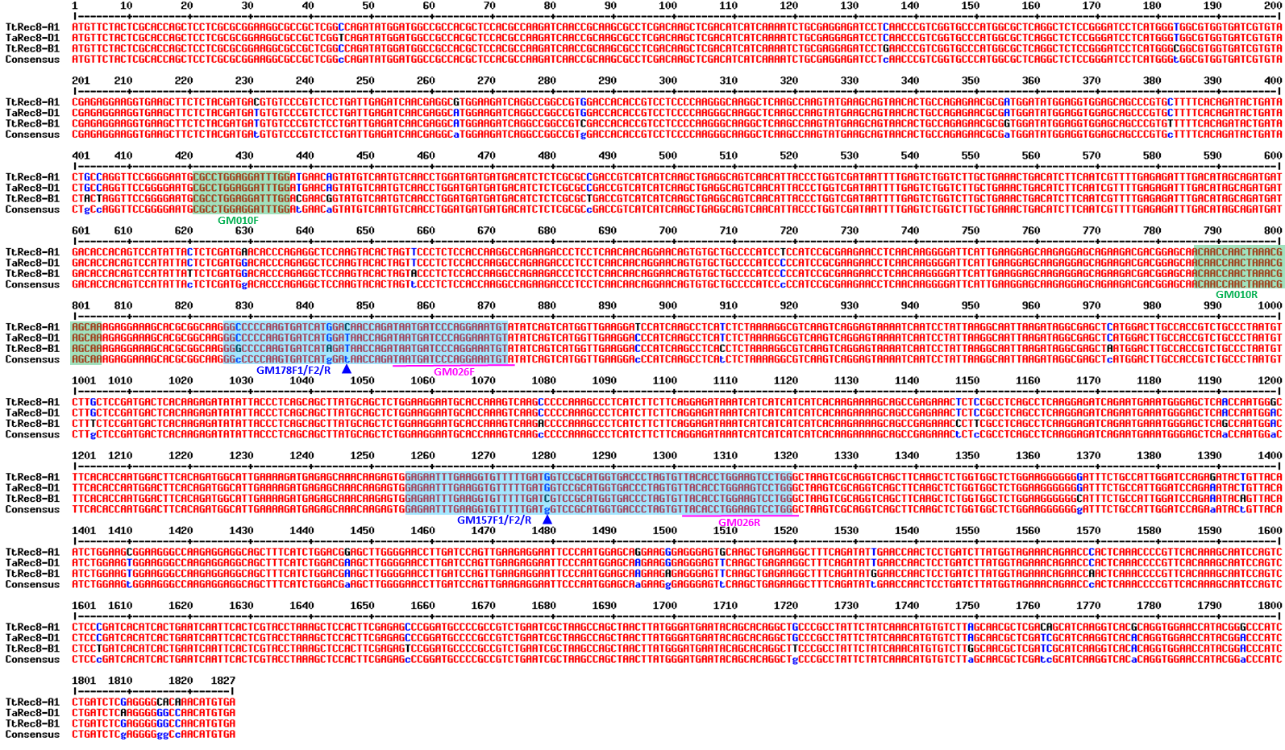
**

**Figure S3.** **CDS alignment of the homoeoalleles *TtRec8-A1*, *TtRec8-B1*, and *TaRec8-D1*.**

The nucleotide differences are indicated in blue and black fonts. The green-highlighted cDNA regions refer to the locations of the primers for real-time PCR (GM010F/GM010R). The light blue-highlighted regions refer to the STARP primer locations (GM157F1/F2/R and GM178F1/F2/R) and their amplicons. The arrow heads point to the SNPs targeted by STARP. The cDNA regions underlined in pink refer to the locations of the primers (GM026F/GM026R) used to amplify the cDNA segment for antibody generation.


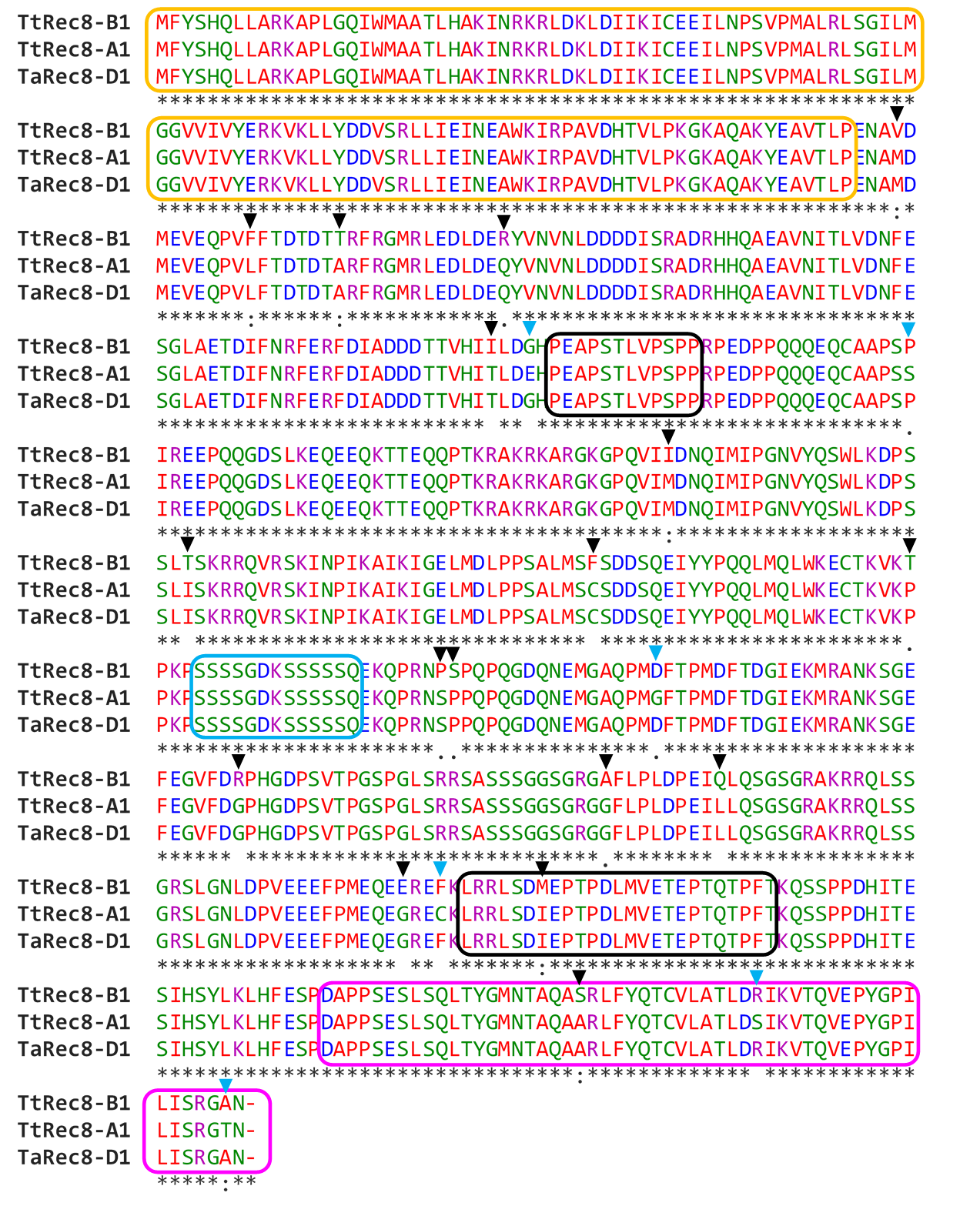


**Figure S4.** **Predicted protein sequences of the wheat *Rec8* homoeoalleles.**

Amino acids are categorized into four groups represented in the letter designations with different colors (AVFPMILW – small + hydrophobic; DE – acidic; RK – basic; STYHCNGQ – hydroxyl + sulfhydryl + amine + G) according to their physicochemical properties. “*”indicates the identical amino acids in the alignment; “:” indicates the amino acids with high similarity; “.” indicates the amino acids with little similarity; blank at the bottom of the alignment means significant difference between the amino acids. Orange and pink boxes demarcate the conserved Rad21/Rec8 domains at the N- and C-terminus (pfam04825 and pfam04824), respectively. Light blue box demarcates the serine-rich region conserved in plant cohesins. Black boxes demarcate the potential PEST motifs. Black arrowheads point to the position where TtRec8-B1 contains an amino acid different from TtRec8-A1 and TaRec8-D1, while blue arrowheads point to the position where TtRec8-A1 contains an amino acid different from TtRec8-B1 and TaRec8-D1.

**
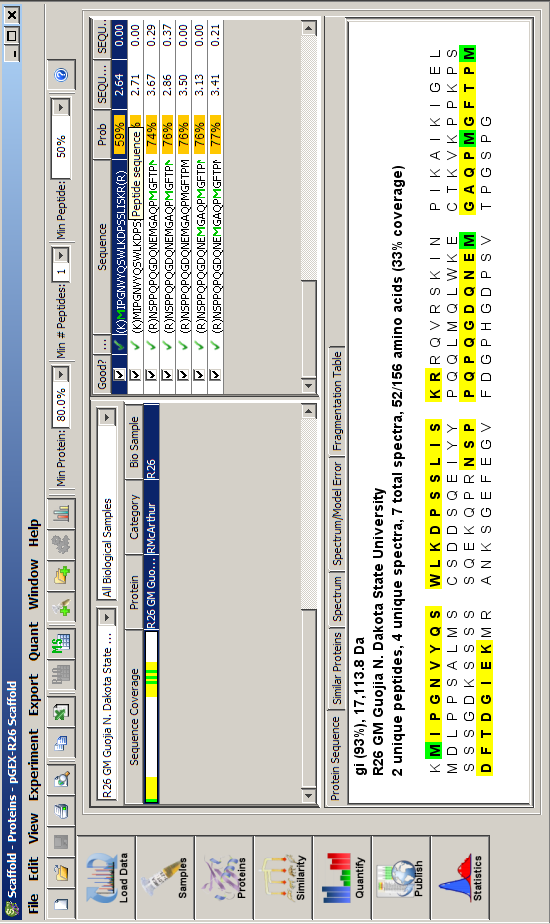
**

Merged

Chromosome

FITC


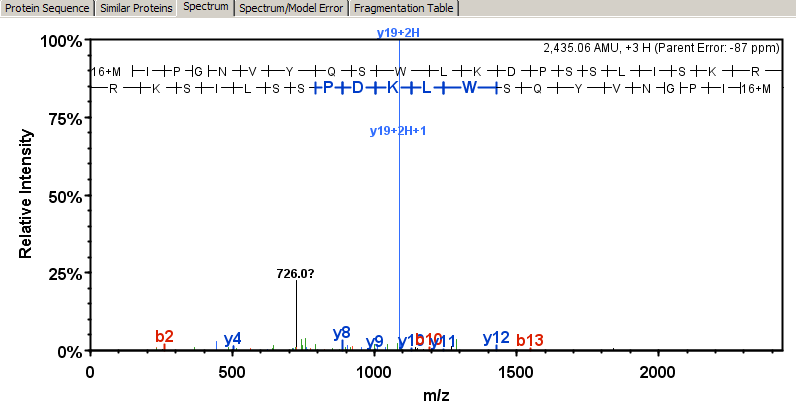


**Figure S5. Verification of polypeptide pGEX-R26 by protein identification assay.**

***Top:*** LC-MS/MS data were collected from the peptide mixture generated by proteolytic digestion of pGEX-R26 polypeptide sample, and the MS/MS spectra were searched against the database of the deduced amino acid component of pGEX-R26. The result showed 33% coverage of the database, as highlighted in yellow. ***Bottom:*** LC-MS/MS spectra of the fragmentation of polypeptide pGEX-R26.

**Table S1. DNA primers and their sequences used in this study.**

| **Primer**  **name** | **Primer sequences** | **Targeted alleles** | **Description** |
| --- | --- | --- | --- |
|  |  |  |  |
| GM157F1 | 5'- CACGACGTTGTAAAACGACGAGAATTTGAAGGTGTTTTTGGTG -3' **(AS-1)*** | *TtRec8-A1*, *TaRec8-D1* | Allele-specific primers for the expression analysis of the wheat *Rec8* homoeoalleles  *TtRec8*-specific primers for cloning and chromosomal localization of *TtRec8*  18S rRNA gene-specific primers for the internal control in real-time PCR  *TtRec8*-specific primers for real-time PCR |
| GM157F2 | 5'- CACGACGTTGTAAAACGACACGACGAGAATTTGAAGGTGTTTTTTATC -3' **(AS-2)** | *TtRec8-B1* |  |
| GM157R | 5'- CCAGGACTTCCAGGTGTA -3' **(Reverse primer)** |  |  |
| GM178F1 | 5'- GCAACAGGAACCAGCTATGACGGCCCCCAAGTGATCATGAAC -3' **(AS-1)** | *TtRec8-A1* |  |
| GM178F2 | 5'- GCAACAGGAACCAGCTATGACATGACGGCCCCCAAGTGATCATAGAT -3' **(AS-2)** | *TtRec8-B1*, *TaRec8-D1* |  |
| GM178R | 5'- ACATTTCCTGGGATCATTATCTGGT -3' **(Reverse primer)** |  |  |
| GM065F | 5'- ACCATACGGGCCCATCCT -3' | *TtRec8-A1*, *TtRec8-B1*, and *TaRec8-D1*  *TtRec8-A1*, *TtRec8-B1*, and *TaRec8-D1* |  |
| GM065R | 5'- ATTCTTTCAGCGTGGCATATCT -3' |  |  |
| GM067F | 5'- CACCTCCTCCTCCGACCT -3' |  |  |
| GM008F | 5'- AAGACCCTCCTCAACAACA -3' |  |  |
| GM008R | 5'- CCTGACTTGACGCCTTTT -3' |  |  |
| GM009F | 5'- AGGCAGTCAACATTACCC -3' |  |  |
| GM009R | 5'- CCTCTTTGCTCGTTTAGTT -3' |  |  |
| PEA-GM003 | 5’- GCAACAGGAACCAGCTATGACGAGGGACTATGGCCGTTTAGG-3’ |  |  |
| GM003 | 5'- GAGGGACTATGGCCGTTTAGG -3' |  |  |
| GM004 | 5'- CACTTCACCGGACCATTCAATCG -3' |  |  |
| GM010F | 5'- CGCCTGGAGGATTTGG -3' |  |  |
| GM010R | 5'- TTGCTCGTTTAGTTGGTTGT -3' |  |  |
| GM026F | 5'- AATA**GAATTC**AAAATGATCCCAGGAAATGTA -3' | *TtRec8-A1*, *TtRec8-B1*, and *TaRec8-D1* | Primers for amplifying the cDNA segment used in antibody generation |
| GM026R | 5'- ACAT**GTCGAC***TCA*CCCAGGACTTCCAGGTGTA -3' |  |  |

*Nucleotides in blue fonts refer to the tail sequence involved in M13/PEA priming for allele detection. Nucleotides in underlined blue fonts are the 5-bp insertion for generating length polymorphism between two alleles. Nucleotides in green fonts are the inserted mismatch nucleotides, and those in red fonts are the SNPs targeted. Nucleotides in bold fonts are the inserted RE recognition sites for *Eco*RI and *Sal*I for cloning purpose, and those in italic brown fonts (TCA) are used to generate a stop codon (TGA) in the expression constructs.

**Table S2. Comparative analysis of the predicted TtRec8-A1 protein with the cohesion proteins from other eukaryotic species**

| **Species** | **Cohesin orthologue** | **GeneID** | **Genbank Accession** | **Similarity with TtRec8-A1** |
| --- | --- | --- | --- | --- |
| *B. distachyon* | Scc1-like | 100824802 | XP_003567819.1 | 80% |
| *O. sativa* | Rad21 | 4339720 | NP_001056426.1 | 68% |
| *Z. mays* | Afd1 | 732730 | NP_001105829.1 | 67% |
| *A.* *thaliana* | Syn1 | 830432 | NP_196168.1 | 42% |
| *D. melanogaster* | Rad21 | 113590 | AAD33593.1 | 40% |
| *X. laevis* | Rad21 | 399129 | AAH97558.1 | 36% |

**Table S3. Genbank accession numbers of the Rec8 orthologues involved in the phylogenetic analysis**

| Species | Cohesin orthologue | Genbank Accession |
| --- | --- | --- |
| *Triticum turgidum* | TtRec8-A1 | MG372313 |
| *Triticum turgidum* | TtRec8-B1 | MG372314 |
| *Triticum aestivum* | TaRec8-D1 | MG372315 |
| *Brachypodium distachyon* | Scc1-like | XP_003567819.1 |
| *Oryza sativa* | Rad21 | NP_001056426.1 |
| *Zea mays* | Afd1 | NP_001105829.1 |
| *Arabidopsis thaliana* | Syn1 | NP_196168.1 |
| *Oryza brachyantha* | Scc1-like | XP_006654842.1 |
| *Setaria italica* | Scc1 | XP_012699922.1 |
| *Sorghum bicolor* | - | XP_002440318.1 |
| *Hordeum vulgare* | - | BAJ93700.1 |
